# Dosimetric comparison of carbon ion radiotherapy and stereotactic body radiotherapy with photon beams for the treatment of hepatocellular carcinoma

**DOI:** 10.1186/s13014-015-0491-8

**Published:** 2015-09-17

**Authors:** Takanori Abe, Jun-ichi Saitoh, Daijiro Kobayashi, Kei Shibuya, Yoshinori Koyama, Hirohumi Shimada, Katsuyuki Shirai, Tatsuya Ohno, Takashi Nakano

**Affiliations:** Department of Radiation Oncology, Gunma University Graduate School of Medicine, 3-39-22, Showa-machi, Maebashi, Gunma 371-8511 Japan; Gunma University Heavy Ion Medical Center, 3-39-22, Showa-machi, Maebashi, Gunma 371-8511 Japan

## Abstract

**Background:**

The purpose of this study was to compare carbon ion radiotherapy (C-ion RT) and stereotactic radiotherapy (SBRT) with photon beams for the treatment of hepatocellular carcinoma (HCC), specifically with regard to the dose volume parameters for target coverage and normal tissue sparing.

**Methods:**

Data of 10 patients who were treated using C-ion RT with a total dose of 60 Gy(RBE) in four fractions were used. The virtual plan of SBRT was simulated on the treatment planning computed tomography images of C-ion RT. Dose volume parameters such as minimum dose covering 90 % of the planning target volume (PTV D90), homogeneity index (HI), conformity index (CI), mean liver dose (MLD), volume of the liver receiving 5 to 60 Gy (V5-60), and max point dose (Dmax) of gastrointestinal (GI) tract were calculated from both treatment plans.

**Results:**

The PTV D90 was 59.6 ± 0.2 Gy(RBE) in C-ion RT, as compared to 56.6 ± 0.3 Gy in SBRT (*p* < 0.05). HI and CI were 1.19 ± 0.03 and 0.79 ± 0.06, respectively in C-ion RT, as compared to 1.21 ± 0.01 and 0.37 ± 0.02, respectively in SBRT. Only CI showed a significant difference between two modalities. Mean liver dose was 8.1 ± 1.4 Gy(RBE) in C-ion RT, as compared to 16.1 ± 2.5 Gy in SBRT (*p* < 0.05). V5 to V50 of liver were higher in SBRT than C-ion RT and significant differences were observed for V5, V10 and V20. Dmax of the GI tract was higher in SBRT than C-ion RT, but did not show a significantly difference.

**Conclusions:**

C-ion RT provides an advantage in both target conformity and normal liver sparing compared with SBRT.

## Background

Hepatocellular carcinoma (HCC) is the sixth most common cancer and third major cause of cancer-related death worldwide [1]. Major causes of HCC are hepatitis virus C, hepatitis virus B and alcohol abuse [2, 3], which may result in hepatic dysfunction in HCC patients at presentation. Surgical resection and percutaneous radiofrequency ablation provide comparable local control rate and overall survival [1, 4]. However, treatment efficacy of both modalities deteriorates in patients with preexisting hepatic dysfunction [5, 6]. For such patients, radiation therapy using photon beams is applied. However, when conventional techniques are used, radiation therapy is limited in its efficacy, due to the difficulty of irradiating the local site while sparing normal liver, which may cause radiation-induced liver disease (RILD) [7]. Stereotactic body radiotherapy (SBRT) with photon beams has been developed with highly precise and conformal beam delivery, which may decrease the dose to the normal liver and provide a less invasive treatment option for patients with hepatic dysfunction [8]. However, it is reported that even with SBRT, treatment efficacy decreases substantially with increasing tumor size [9, 10]. Particle beam radiotherapy such as proton and carbon ion radiotherapy (C-ion RT) was applied for HCC due to their physical advantages that deposit maximum energy sharply, with a penetration range. The advantage of particle beam RT enables the delivery of an adequate dose to the tumor while minimizing the dose to surrounding normal tissue [11]. Proton therapy and C-ion RT for HCC were reported to be effective and well tolerated for advanced tumors [12–14]. In addition, carbon ion beams have a higher relative biological effect due to larger mean linear energy transfer [15]. However, no previous reports have compared dose volume parameters for C-ion RT versus SBRT with regard to target volume coverage and sparing of organ at risk. Therefore, the purpose of this study was to compare the dose volume parameters to the target and organ at risk using C-ion RT and SBRT in patients with HCC.

## Methods

### Patients

Since 2012, HCC patients have been treated with C-ion RT at our institution. With approval from the institutional review board of Gunma University Hospital, data from 10 consecutive HCC patients were used for this study.

### Carbon ion radiotherapy

Patients received 60.0 Gy(RBE) in four fractions of C-ion RT. In this study, the dose of C-ion RT is expressed as Gy(RBE), which was calculated by multiplying the carbon physical dose (Gy) by the RBE. The RBE for therapeutic carbon beams is assumed to be three at a neutron-equivalent depth of therapeutic carbon beams, which is very near the distal end of the spread-out Bragg peak (SOPB) [15, 16]. Carbon ion beams were generated by the heavy particle accelerator at GHMC. For HCC treatment, passive scattering technique was applied. Energies of the accelerated beams were 290 MeV/u, 380 MeV/u, and 400 MeV/u. Beam energy was decided according to the depth of the tumor. The SOBP is created by the ridge filter. Beam range is adjusted by a range shifter and range compensator. C-ion RT plans were calculated using the XiO-N (ELEKTA, Stockholm, Kingdom of Sweden and Mitsubishi Electric, Tokyo, Japan). The XiO-N is a XiO (ELEKTA)-based treatment planning system consisting of external dose calculation engine “k2 Dose” and a source data management tool (Mitsubishi Electric, Tokyo, Japan). Immobilization devices such as fixation cushions and thermoplastic shells (3 mm thickness) were used to acquire treatment planning computed tomography (CT) images. After immobilization, respiratory-gated CT images were acquired. For treatment planning, images of the expiratory phase were used. The gross tumor volume (GTV) was defined on treatment planning CT by referring to the fused CT image with a contrast material at the arterial phase. The clinical target volume (CTV) margin, including subclinical disease invasion, was added to the GTV, adding 5 mm in all directions (excluding the chest wall). The internal margin (IM) was determined by the tumor motion demonstrated in 4-D CT images to encompass residual respiratory tumor motion. The planning target volume (PTV) included the CTV, IM, and setup margin (3 mm). The Gating window was set to 30 % of the expiratory phase to 30 % of the inspiratory phase to minimize the influence of tumor respiratory motion. Planning are described in a previous study [17].

### Treatment planning of stereotactic body radiotherapy

The treatment plan of 60 Gy in four fractions of SBRT was simulated on the CT images of patients who received C-ion RT. Planning aim was to cover the PTV for at least 90 % of the prescribed dose. Treatment plan were calculated by treatment planning system of the photon therapy (Eclipse, Varian Medical Systems, Inc. California, USA). All the contours were the same as those of the C-ion RT. SBRT plans employed seven coplanar 10 MV photon beams with an even distribution of gantry angles with avoiding gastrointestinal (GI) tract and spinal cord. The weights of each field were also arranged to cover the PTV for at least 90 % of the prescribed dose and minimize the dose to the OARs. All treatment plans for C-ion RT were created by medical physicists and delivered to patients. All SBRT plans were created by single radiation oncologist for this study.

### Dose volume parameters

The following dose volume parameters were assessed for PTV; minimum dose covering 90 % of the planning target volume (PTV D90), homogeneity index (HI; maximum dose/minimum dose in the target), and conformity index (CI; volume receiving the prescribed dose/target volume) [18]. To compare normal tissue sparing, the following dose volume parameters were assessed for normal liver and GI tract. Normal liver was defined as liver except GTV. Mean liver dose (MLD), normal liver volumes receiving more than 5 Gy(RBE) (V5), 20 Gy(RBE) (V20), 30 Gy(RBE) (V30), 40 Gy(RBE) (V40), 50 Gy(RBE) (V50), and 60 Gy(RBE) (V60), and maximum point dose (D_max_) to the GI tract were calculated. Mean parameters in the two modalities were compared by Student’s *t*-test and *P* < 0.05 was considered statistically significant. All statistical analyses were performed using IBM SPSS Statistics for Windows, Version 21.0 (SPSS Inc., Armonk, NY, USA).

## Results

### Patient characteristics

Ten patients were included in this study. Tumor sites were as follows: S1 (1 patient), S4 (2 patients), S6 (1 patient), S7 (4 patients), and S8 (2 patients). T stages were as follows: T1 (8 patients), T2 (1 patient), and T3 (2 patients). The mean diameter of the GTV was 4.8 cm (range; 1.4 – 8.0 cm) and mean values for the GTV and PTV were 40.3 cm3 (range; 0.69 – 151.1 cm3) and 110.8 cm3 (range; 12.4 – 321.2 cm3), respectively.

### Dose volume parameters

**Fig. 1:**
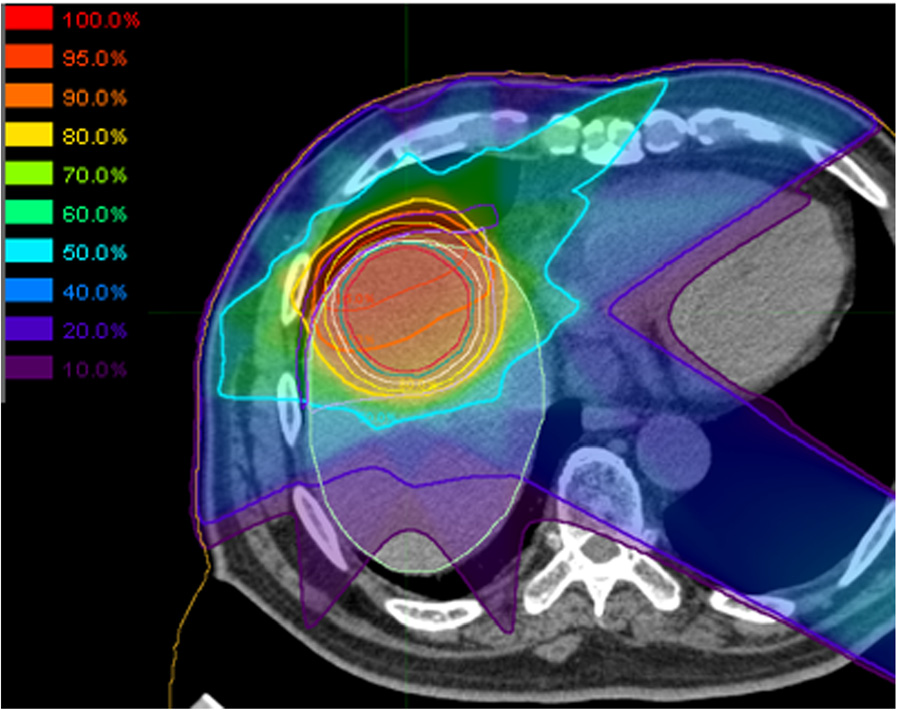
Representative dose distribution of stereotactic body radiotherapy. The thick light blue line shows the 30 Gy isodose line

**Fig. 2:**
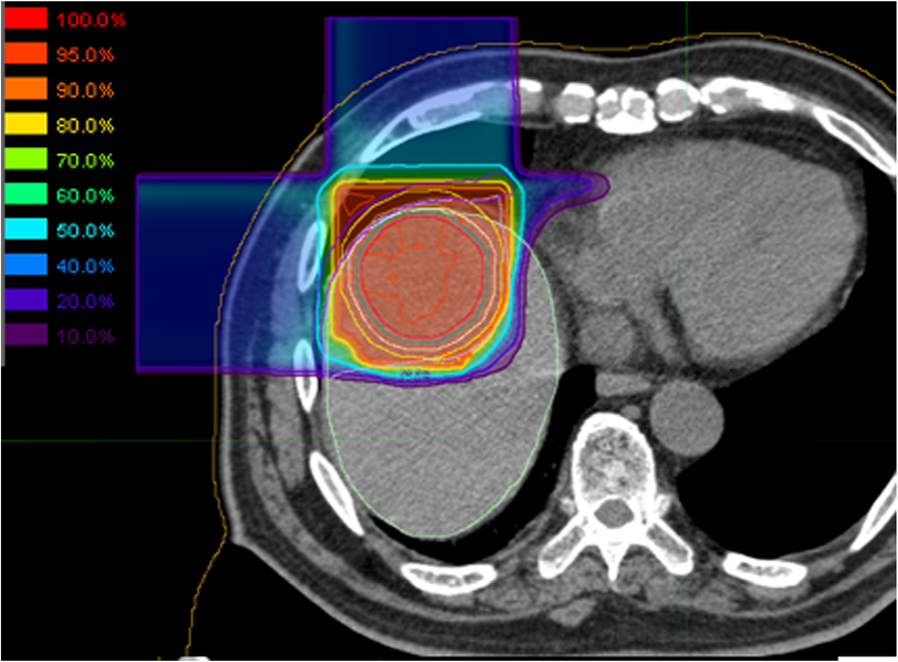
Representative dose distribution of carbon-ion radiotherapy. Dose to the normal tissue is less than that in SBRT

**Fig. 3:**
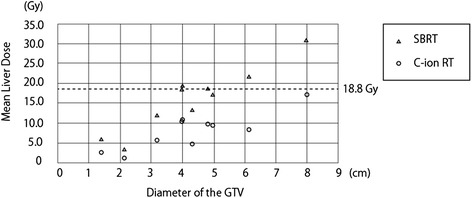
Scatter plot diagram of the diameters of the gross tumor volume and mean liver dose. Stereotactic body radiotherapy could not achieve the dose constraints for normal liver (mean liver dose > 18.8 Gy) in four patients for whom the tumor diameter was more than 4 cm

Representative dose distributions of SBRT and C-ion RT are shown in Figs. 1 and 2. Dosimetric parameters of C-ion RT and SBRT were calculated from both treatment plans and summarized in Table 1. The PTV D90 was 59.6 ± 0.2 Gy(RBE) for C-ion RT, as compared to 56.6 ± 0.3 Gy for SBRT, indicating a significant difference (*p* < 0.05). The HI and CI were 1.19 ± 0.03 and 0.79 ± 0.06, respectively, for C-ion RT, as compared to 1.21 ± 0.01 and 0.37 ± 0.02, respectively, for SBRT; only CI showed a significant difference. MLD for normal tissue sparing was 8.1 ± 1.4 Gy(RBE) for C-ion RT, as compared to 16.1 ± 2.5 Gy for SBRT (*p* < 0.05). The relationship between MLD and GTV diameter is shown in Fig. 3. V5 to V50 were higher in SBRT than C-ion RT, while significant differences were observed for V5, V10, and V20. Dmax of the GI tract was higher in SBRT than C-ion RT, but did not constitute a significant difference.

**Table 1:** Dosimetric parameters

	Carbon ion radiotherapy	SBRT with photons	*P* value
PTV D90	59.6 ± 0.2 (GyRBE)	56.6 ± 0.3 (Gy)	*p* < 0.05
HI	1.19 ± 0.03	1.21 ± 0.01	*p* = 0.61
CI	0.79 ± 0.06	0.37 ± 0.02	*p* < 0.05
Mean liver dose	8.1 ± 1.4 (GyRBE)	16.1 ± 2.5 (Gy)	*p* < 0.05
Liver V5	20.2 ± 3.2 %	53.8 ± 7.2 %	*p* < 0.05
Liver V20	14.2 ± 2.5 %	31.5 ± 6.2 %	*p* < 0.05
Liver V30	11.6 ± 2.3 %	19.7 ± 4.1 %	*p* = 0.11
Liver V50	8.5 ± 1.8 %	9.7 ± 2.1 %	*p* = 0.67
D max of GI tract	8.4 ± 4.3 (GyRBE)	17.4 ± 7.1 (Gy)	*p* = 0.29

*Abbreviations*: *PTV D90* Minimum dose covering the 90 % of planning target volume, *HI* Homogeneity index, *CI* Conformity index, *V5-50* Volume of liver received more than 5 Gy to 50 Gy, *GI tract* Gastrointestinal tract

## Discussion

In this study, PTV D90 and CI showed significant differences between C-ion RT and SBRT while HI showed no significant difference. CI was significantly higher in C-ion RT, which indicates the superiority of C-ion RT for its ability to generate more conformal dose distribution than SBRT. The physical characteristics of the carbon ion beam, specifically, the distal fall-off of the Bragg peak and less lateral scatter contributed to the more conformal and homogeneous dose delivery to the target than SBRT.

Many reports have analyzed the relationship between dosimetric parameters and RILD [19–21]. Tse et al reported that no RILD requiring treatment occurred in the patient for whom the MLD was less than 22 Gy with a total dose of 24 – 60 Gy in 6 fractions of hypofractionated radiotherapy [20]. A MLD of 22 Gy in 6 fractions is equal to 18.8 Gy(RBE) in four fractions, as calculated by the linear quadratic equation model assuming an α/β ratio of 3 for normal liver [22]. In our study, SBRT could not achieve this dose constraint (MLD > 18.8 Gy) in four of ten patients, while, C-ion RT could achieve the constraint in all patients, even though there were some bulky tumors. In this study, the dose constraint could not be satisfied for tumors with diameters of more than approximately 4 cm with SBRT, which was shown in (Fig. 3). This suggests that C-ion RT has advantage in treating patients with bulky tumors. Pan et al. reported that MLD should be less than 6 Gy in patients with poor liver function case (Child-Pugh score B) [19]. In our study, low dose volume for a normal liver (e.g., V5-20) was significantly lower for C-ion RT than SBRT, and the difference in DVH parameters between the two modalities tended to be bigger in lower doses. Thus, C-ion RT may have advantage for the patients with poor liver function, due to its capacity to decrease low dose scattering to the normal liver.

Dose volume parameters to the GI tract such as the duodenum and stomach showed no significant differences between the two modalities in this study. With our protocol of C-ion RT for the treatment of HCC with four fractions, patients with tumor located less than 1 cm from the GI tract were not eligible due to the risk of late complications such as ulceration and fistula. Elimination of these patients may have let to the results that no significant difference was observed in dose to the GI tract between the two modalities. However, D0.5 cm^3^ – D2cm^3^ and Dmax to GI tract tended to be twice as high for SBRT than C-ion RT, although the difference was not significant. In our new protocol for HCC locating adjacent GI tract, total dose and fractionation were modified to 64.8 Gy(RBE) in 12 fractions considering the tolerance dose to the GI tract. This new protocol may allow for significantly more sparing of the GI tract

The present study has some limitations which must be noted. First, the RBE of carbon ion beams may be influenced by fraction dose, clinical endpoint, and other factors, all of which may affect our results. In Japan where a beam-scattering method with a passive beam delivery system is used, the dose distributions of therapeutic carbon ion beams are determined by in-vitro Human Salivary Gland (HSG) cell survival response and by clinical experience from fast neutron radiotherapy in National Institute of Radiological Sciences (NIRS). Moderate radiosensitivity of HSG cells is expected to be a typical tumor response to carbon ion beams. Initially, the biological dose distribution is designed in order to cause a flat biological effect on HSG cells in the SOBP region. Then, the entire biological dose distribution is increased evenly to attain an RBE of 3.0 at a depth where dose-averaged LET (linear energy transfer) is 80 keV/mum. At that point, biological experiments have shown that carbon ions can be expected to have a biological effect identical to that of fast neutrons, which show a clinical RBE of 3.0 for fast neutron radiotherapy at NIRS [16]. However, optimum dose-fractionation schedules of C-ion RT, (60 Gy(RBE) in four fractions) for HCC have been established through clinical experience, including extensive dose escalation studies at NIRS using the above RBE model [14]. On the other hand, Kang et al and Jang et al reported favorable results with 60 Gy in three fractions of X-ray SBRT for HCC [23, 24]. While the optimal dose to HCC in X-ray SBRT is not established, 60 Gy in four fractions can be applied in clinical settings. Therefore, we think that 60 Gy in four fractions of X-ray SBRT could be candidate to compare against of 60 Gy(RBE) in four fractions of C-ion RT for this dosimetric comparative study. Second, this was a mono-institutional study, and thus policy of the planner could have produced some subjective bias. Treatment plans for each modality were made by staff members of varying experience levels who were blinded to each other. Both of the planners were also blinded to the clinical results of C-ion RT to reduce bias as much as possible.

## Conclusions

In conclusion, C-ion RT for HCC provides an advantage in both target conformity and normal liver sparing compared with SBRT

## References

[1] Forner A, Llovet JM, Bruix J (2012). Hepatocellular carcinoma. Lancet.

[2] Umemura T, Ichijo T, Yoshizawa K, Tanaka E, Kiyosawa K (2009). Epidemiology of hepatocellular carcinoma in Japan. J Gastroenterol.

[3] Bruno S, Silini E, Crosignani A, Borzio F, Leandro G, Bono F (1997). Hepatitis C virus genotypes and risk of hepatocellular carcinoma in cirrhosis: a prospective study. Hepatology.

[4] Llovet JM, Bruix J (2003). Systematic review of randomized trials for unresectable hepatocellular carcinoma: Chemoembolization improves survival. Hepatology.

[5] Cho YK, Kim JK, Kim MY (2009). Systematic review of randomized trials for hepatocellular carcinoma treated with percutaneous ablation therapies. Hepatology.

[6] Arii S, Yamaoka Y, Futagawa S, Inoue K, Kobayashi K, Kojiro M (2000). Results of surgical and nonsurgical treatment for small-sized hepatocellular carcinomas: A retrospective and nationwide survey in Japan. The Liver Cancer Study Group of Japan. Hepatology.

[7] Lawrence TS, Ten Haken RK, Kessler ML, Robertson JM, Lyman JT, Lavigne ML (1992). The use of 3-D dose volume analysis to predict radiation hepatitis. Int J Radiat Oncol Biol Phys.

[8] Bujold A, Massey CA, Kim JJ, Brierley J, Cho C, Wong RK (2013). Sequential phase I and II trials of stereotactic body radiotherapy for locally advanced hepatocellular carcinoma. J Clin Oncol.

[9] Kimura T, Aikata H, Takahashi S, Takahashi I, Nishibuchi I, Doi Y (2015). Stereotactic body radiotherapy for patients with small hepatocellular carcinoma ineligible for resection or ablation therapies. Hepatol Res.

[10] Blomgren H, Lax I, Naslund I, Svanstrom R (1995). Stereotactic high dose fraction radiation therapy of extracranial tumors using an accelerator. Clinical experience of the first thirty-one patients. Acta Oncol.

[11] Ohno T (2013). Particle radiotherapy with carbon ion beams. EPMA J.

[12] Hata M, Tokuuye K, Sugahara S, Fukumitsu N, Hashimoto T, Ohnishi K (2006). Proton beam therapy for hepatocellular carcinoma with limited treatment options. Cancer.

[13] Chiba T, Tokuuye K, Matsuzaki Y, Sugahara S, Chuganji Y, Kagei K (2005). Proton beam therapy for hepatocellular carcinoma: a retrospective review of 162 patients. Clin Cancer Res.

[14] Kato H, Tsujii H, Miyamoto T, Mizoe JE, Kamada T, Tsuji H (2004). Results of the first prospective study of carbon ion radiotherapy for hepatocellular carcinoma. Int J Radiat Oncol Biol Phys.

[15] Kanai T, Endo M, Minohara S, Miyahara N, Koyama-ito H, Tomura H (1999). Biophysical characteristics of HIMAC clinical irradiation system for heavy ion radiation therapy. Int J Radiat Oncol Biol Phys.

[16] Matsufuji N, Kanai T, Kanematsu N, Miyamoto T, Baba M, Kamada T (2007). Specification of carbon ion dose at the National Institute of Radiological Sciences (NIRS). J Radiat Res.

[17] Tashiro M, Ishii T, Koya J, Okada R, Kurosawa Y, Arai K (2013). Technical approach to individualized respiratory-gated carbon-ion therapy for mobile organs. Radiol Phys Technol.

[18] Feuvret L, Noël G, Mazeron JJ, Bey P (2006). Conformity index: a review. Int J Radiat Oncol Biol Phys.

[19] Pan CC, Kavanagh BD, Dawson LA, Li XA, Das SK, Miften M (2010). Radiation-associated liver injury. Int J Radiat Oncol Biol Phys.

[20] Tse RV, Hawkins M, Lockwood G, Kim JJ, Cummings B, Knox J (2008). Phase I study of individualized stereotactic body radiotherapy for hepatocellular carcinoma and intrahepatic cholangiocarcinoma. J Clin Oncol.

[21] Liang SX, Zhu XD, Xu ZY, Zhu J, Zhao JD, Lu HJ (2006). Radiation-induced liver disease in three-dimensional conformal radiation therapy for primary liver carcinoma: The risk factors and hepatic radiation tolerance. Int J Radiat Oncol Biol Phys.

[22] Fowler JF (1989). The linear-quadratic formula and progress in fractionated radiotherapy. Br J Radiol.

[23] Kang JK, Kim MS, Cho CK, Yang KM, Yoo HJ, Kim JH (2012). Stereotactic body radiation therapy for inoperable hepatocellular carcinoma as a local salvage treatment after incomplete transarterial chemoembolization. Cancer.

[24] Jang WI, Kim MS, Bae SH, Cho CK, Yoo HJ, Seo YS (2013). High-dose stereotactic body radiotherapy correlates increased local control and overall survival in patients with inoperable hepatocellular carcinoma. Radiat Oncol.

